# Verbal Short-Term Memory Disturbance in the Primary Progressive Aphasias: Challenges and Distinctions in a Clinical Setting

**DOI:** 10.3390/brainsci11081060

**Published:** 2021-08-12

**Authors:** David Foxe, Sau Chi Cheung, Nicholas J. Cordato, James R. Burrell, Rebekah M. Ahmed, Cathleen Taylor-Rubin, Muireann Irish, Olivier Piguet

**Affiliations:** 1 School of Psychology, The University of Sydney, Sydney, NSW 2006, Australia; david.foxe@sydney.edu.au (D.F.); sau.cheung@sydney.edu.au (S.C.C.); muireann.irish@sydney.edu.au (M.I.); 2 Brain and Mind Centre, The University of Sydney, Sydney, NSW 2050, Australia; Nicholas.Cordato@health.nsw.gov.au (N.J.C.); james.burrell@sydney.edu.au (J.R.B.); rebekah.ahmed@sydney.edu.au (R.M.A.); 3 St George Clinical School, University of New South Wales, Sydney, NSW 2217, Australia; 4 The Department of Aged Care, St George Hospital, Sydney, NSW 2217, Australia; 5 Calvary Health Care Kogarah, Sydney, NSW 2217, Australia; 6 Concord Clinical School, Sydney Medical School, The University of Sydney, Sydney, NSW 2139, Australia; 7 Central Medical School, The University of Sydney, Sydney, NSW 2006, Australia; 8 Department of Cognitive Science, Faculty of Medicine, Health and Human Sciences, Macquarie University, Sydney, NSW 2109, Australia; Cathleen.Taylor@health.nsw.gov.au; 9 Department of Speech Pathology, Uniting War Memorial Hospital, Sydney, NSW 2024, Australia

**Keywords:** primary progressive aphasia, frontotemporal dementia, Alzheimer’s disease, neuropsychology, span, sentence repetition, working memory, phonological, visuospatial

## Abstract

Impaired verbal ‘phonological’ short-term memory is considered a cardinal feature of the logopenic variant of primary progressive aphasia (lv-PPA) and is assumed to underpin most of the language deficits in this syndrome. Clinically, examination of verbal short-term memory in individuals presenting with PPA is common practice and serves two objectives: (i) to help understand the possible mechanisms underlying the patient’s language profile and (ii) to help differentiate lv-PPA from other PPA variants or from other dementia syndromes. Distinction between lv-PPA and the non-fluent variant of PPA (nfv-PPA), however, can be especially challenging due to overlapping language profiles and comparable psychometric performances on verbal short-term memory tests. Here, we present case vignettes of the three PPA variants (lv-PPA, nfv-PPA, and the semantic variant (sv-PPA)) and typical Alzheimer’s disease (AD). These vignettes provide a detailed description of the short-term and working memory profiles typically found in these patients and highlight how speech output and language comprehension deficits across the PPA variants differentially interfere with verbal memory performance. We demonstrate that a combination of verbal short-term and working memory measures provides crucial information regarding the cognitive mechanisms underlying language disturbances in PPA. In addition, we propose that analogous visuospatial span tasks are essential for the assessment of PPA as they measure memory capacity without language contamination.

## 1. Introduction

Impaired verbal ‘phonological’ short-term memory is considered a cardinal feature of the logopenic variant of primary progressive aphasia (lv-PPA) and is thought to underpin many of the language deficits in this syndrome [1]. Indeed, lv-PPA patients display impaired digit, letter, and word span on formal testing but perform normally on single-digit and -word repetition tasks [2,3,4]. Importantly, these deficits occur in the context of relatively preserved grammar and articulation, although phonological paraphasias may be present [1,3]. Poor verbal short-term memory performance also occurs in the non-fluent variant (nfv-PPA), although this impairment is typically due to motor speech or articulatory deficits [1,3,4]. In contrast, verbal short-term memory performance remains relatively spared in the early stages of the semantic variant of PPA (sv-PPA) [3,4]. These distinct verbal short-term memory profiles led the international consensus criteria for PPA to include ‘impaired sentence repetition and phrases’ as a core clinical feature of lv-PPA [1]—prompting clinicians to evaluate the verbal short-term memory system when assessing patients with a differential diagnosis of lv-PPA.

Despite these recommendations, multiple challenges exist for clinicians assessing these skills at the individual case level. For example, differentiating lv-PPA from nfv-PPA hinges on detecting motor speech and/or grammatical errors—a skill which requires considerable expertise in language assessment [5]. In addition, the presence and severity of these speech and language features are variable, especially in the early stages of the disease, making the distinction between lv-PPA and nfv-PPA challenging [6,7,8,9,10].

In clinical practice, the combination of language and short-term memory tests, however, can improve the clinician’s ability to detect phonological impairment and delineate lv-PPA from the other PPA variants [3,11]. Evaluation of performance scores across tests, as well as awareness of the qualitative aspects of language (e.g., phonological disturbance, dysarthria, agrammatism), helps determine if impaired performance on verbal short-term memory measures is due to the breakdown of the verbal store and rehearsal system—indicating lv-PPA—or to a breakdown of other processes (e.g., nfv-PPA: motor speech programming deficits; sv-PPA: disrupted conceptual knowledge resulting in poor understanding/recollection of words or phrases) [3,11,12]. While these views are well-documented in PPA group comparison studies, attempts to implement this understanding through specific tests at an individual patient level have been limited. Investigations at the case level have several advantages over larger PPA group comparison studies, including the ability to: (i) interpret individual cases based on established norms tailored to the age and education of the individual; (ii) establish a differential diagnosis without referencing a demographic and disease severity matched PPA sample group, and; (iii) place emphasis on interpreting important qualitative aspects of language in conversational speech and on formal standardised testing.

In this study, we explored in detail the short-term memory profiles of individual patients with PPA (lv-PPA, nfv-PPA, sv-PPA) and, for comparison, Alzheimer’s disease (AD). Using tests typically administered in secondary and tertiary clinics, we demonstrate how the language deficits of each PPA variant influence performance across various measures of verbal short-term memory and working memory. We also highlight how the breakdown of these performances can provide clinicians with qualitative insights into the core speech and memory mechanisms affected in an individual patient. Finally, we propose that the assessment of visuospatial short-term and working memory is relevant for the establishment of an accurate diagnosis of PPA.

## 2. Materials and Methods

The four patients presented here as case vignettes were seen at the FRONTIER Frontotemporal Dementia Research Group at the Brain and Mind Centre, The University of Sydney. They all underwent a comprehensive neurological (NJC, JRB, RMA), and systematic cognitive (DF, SCC) and speech assessment (CTR, DF, SCC), as well as structural brain magnetic resonance imaging (MRI). Sentence repetition was phonetically transcribed by CTR and qualitatively scored using the Hohlbaum, Dressel [12] scoring criteria. This study was approved by the Human Research Ethics Committee of the South-Eastern Sydney Local Area Health District (HREC 10/126). All participants provided written informed consent in accordance with the Declaration of Helsinki. Patient initials have been altered to protect the privacy of the individuals and their families.

## 3. Case Vignettes

### 3.1. lv-PPA Patient: NS

At presentation, NS was a 67-year-old, right-handed man (Table 1). He had 12 years of education and had been retired for 7 years, having previously worked in government services and in the tourism sector. He had also been heavily involved in managing the finances and building repairs at his local church but had ceased these duties approximately two years prior to his visit. His past medical history revealed a coronary stent 3 years prior to the assessment, and high cholesterol which was managed with medication. There was no known family history of dementia or other neurodegenerative conditions.

NS was assessed following a 4-year history of speech and language difficulties. Initial symptoms included mispronouncing some words (“stunt” for ‘stent’; “wiltered” for ‘withered’), word substitutions, slowed reading rate, surface dyslexia, and spelling errors. Word-finding difficulties had reportedly become more apparent in the 2 years prior to the visit, particularly in instances that required rapid spontaneous speech. Cognitively, NS felt less confident about his memory and concentration. He also experienced some topographical disorientation in unfamiliar locations. No other cognitive or motor changes were reported, and he remained independent in all activities of daily living (ADLs). His wife had not noticed any behavioural or personality changes and there was no history of psychiatric features. NS did not report any symptoms of depression, anxiety, or stress, and he demonstrated appropriate emotional reactivity during the assessment.

#### 3.1.1. Neuropsychological Assessment

**Figure 1:**
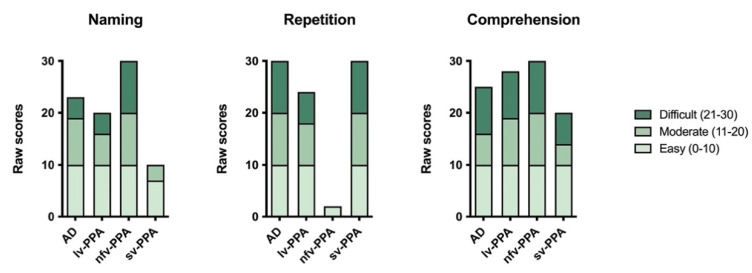
Raw scores on the Naming, Repetition, and Comprehension subtests of the Sydney Language Battery. Note, due to speech output difficulties, the nfv-PPA patient wrote their responses for the Naming subtest (Appendix A).

Based on his educational and vocational history, NS’s estimated premorbid level of functioning was average. On a measure of general cognitive ability, the Addenbrooke’s Cognitive Examination-III (ACE-III), he scored 66/100 which was well-below normal limits (normal > 88), [13,25] (Table 1). His conversational speech was dysfluent with frequent word-finding pauses and phonological errors, though prosody was intact. Formal neuropsychological assessment revealed moderate to severe expressive language difficulties. Verbal fluency and confrontation naming were very impaired, and repetition of multisyllabic words was also reduced somewhat (Table 1, Figure 1). In contrast, comprehension (i.e., word-picture matching) and conceptual semantic knowledge were relatively preserved (Figure 1).

**Figure 2:**
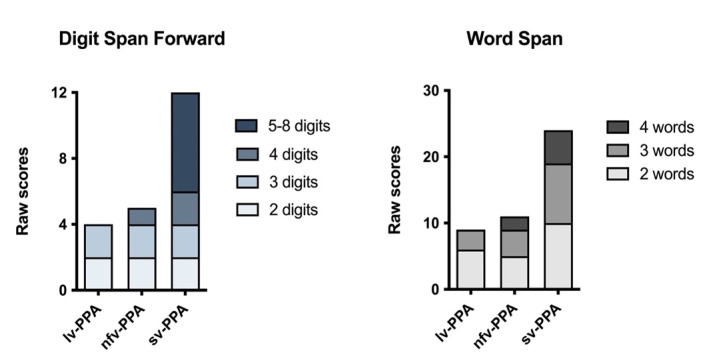
Raw scores on the Digit Span Forward and Word Span tests. The gradient colours/shading within the bar charts represent the gradient levels of difficulty (i.e., Digit Span: raw scores at the 2–, 3–, 4–, and 5–8-digit item levels; Word Span: raw scores at the 2–, 3–, and 4–word item levels).

**Figure 3:**
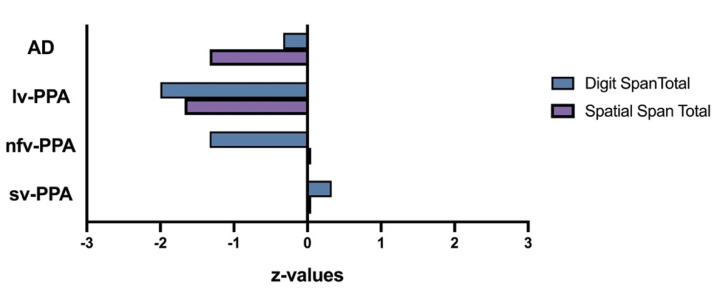
Digit and Spatial Span total raw scores graphically represented as age-adjusted z-values. Overall performance on Digit Span was very impaired for the lv-PPA patient, borderline-impaired for the nfv-PPA patient, and average for the sv-PPA and AD patients. Overall performance on Spatial Span was borderline-impaired for the lv-PPA and AD patients, and average for the nfv-PPA and sv-PPA patients. Overall Digit Span was significantly (0.05) worse than Spatial Span for the nfv-PPA patient; with the reverse pattern (i.e., Spatial < Digits) found for the AD patient. There was no statistical difference between test modality for lv-PPA and sv-PPA patients (statistical thresholds were taken from Table F4 [Appendix F] of the WMS III Scoring Manual).

Verbal short-term memory (i.e., Digit Span Forward, Sentence Repetition, Word Span) and verbal working memory (i.e., Digit Span Backward) were extremely impaired (Table 1 and Table 2, Figure 2). His performance on the visuospatial counterpart tasks was comparatively better although still fell in the borderline-impaired range (Table 1, Figure 3).

**Figure 4:**
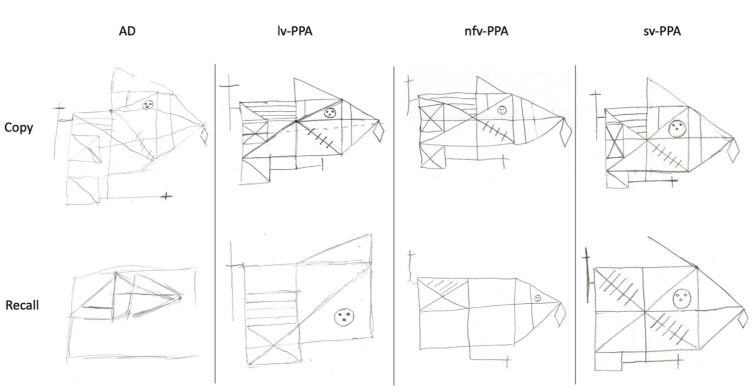
Rey Complex Figure copy and 3-minute recall. Copy performance was very impaired for the AD patient, borderline-impaired for the lv-PPA and nfv-PPA patients, and within normal limits for the sv-PPA patient. Three-minute recall performance was very impaired for the AD patient, borderline-impaired for the lv-PPA patient, and within normal limits for the nfv-PPA and sv-PPA patients.

Executive functioning difficulties were also evident. Specifically, NS demonstrated impairments in proverb interpretation, and rapid set-shifting. Complex visuo-constructional planning was disorganised and extremely fragmented (Figure 4) in the context of intact basic visuo-perceptual skills and psychomotor speed. This was likely to have impacted on his visual memory performance which was borderline-impaired.

#### 3.1.2. Clinical Opinion about the Patient’s Verbal and Visuospatial Short-Term and Working Memory Profile

NS demonstrates the hallmark verbal short-term memory disorder characteristic of lv-PPA, evidenced by impaired digit span, word span, and sentence repetition, in the context of relatively spared repetition of high frequency (i.e., lower cognitive load) multisyllabic words. Qualitatively, NS’s intact repetition of multisyllabic words but impaired sentence repetition suggests that the latter arises from difficulties accessing and rehearsing verbal information in mind—that is, a dissolution of the verbal short-term memory system, rather than from deficits in motor speech production (Table 2) [11,12]. His mild articulatory errors and adequate prosody during speech, with well-articulated sound substitutions and lack of distortions further supports this position. Notably, NS’s short-term and working memory impairments appear to extend beyond the verbal domain, evidenced by his impaired visuospatial span.

#### 3.1.3. Brain MRI and Clinical Diagnosis

**Figure 5:**
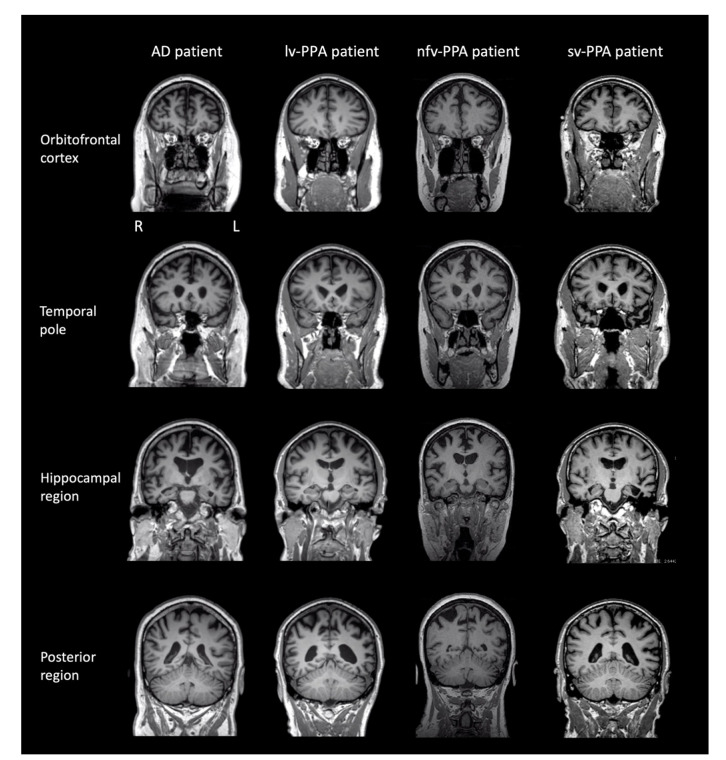
Brain T1 magnetic resonance images (MRI) of patients at the time of neuropsychological testing. Note: L = left; R = right. Brain images are presented in the coronal plane. lv-PPA patient: Mild generalised cortical atrophy was evident, slightly more prominent on the left than the right, extending posteriorly to involve the parietal lobes. There was marginally greater atrophy in the peri-insular region on the left than the right. nfv-PPA patient: Mild generalised cortical atrophy with particular involvement of the left peri-insular region anteriorly. sv-PPA patient: Severe atrophy of the anterior temporal pole bilaterally, but much worse on the left than the right. AD patient: Mild-moderate generalised cortical atrophy with involvement of the mesial temporal lobes.

T1 coronal brain MR images revealed mild generalised cortical atrophy, slightly more prominent on the left than the right, extending posteriorly to involve the parietal lobes (Figure 5). There was marginally greater atrophy in the peri-insular region on the left than the right. T2 weighted MRI images showed occasional hyperintensities in the cerebral hemispheres which were within normal limits for NS’s age. The pattern of brain atrophy, clinical history, language, and neuropsychological profile were consistent with a diagnosis of lv-PPA (Table 3a). Pathological confirmation was unavailable as NS is still alive.

### 3.2. nfv-PPA Patient: ML

At presentation, ML was a 64-year-old, right-handed woman with 12 years of education (Table 1). She had been retired for five years, having previously worked as a shop owner and a public servant. She had a past history of liver disease due to hepatitis C, hepatic cirrhosis, and long-term alcohol consumption. At the time of the assessment, ML had been abstinent from alcohol for 7 years. Her father had been diagnosed with dementia (type unknown) in his 80s and died at the age of 87.

ML was seen following a 5-year history of progressively deteriorating speech which had worsened noticeably over the last 12 months. Initial symptoms also included frequent spelling errors and incorrect sentence construction. Her husband reported that her text messages often had errors but remained largely understandable. She reported occasional Yes/No and Hi/Bye confusion but had no trouble using corresponding non-verbal gestures. No other cognitive changes were reported. She described no swallowing difficulties and no Parkinsonian symptoms. She did not report any symptoms of depression, anxiety, or stress on a self-report measure of recent mood, and she demonstrated appropriate emotional reactivity during the assessment. According to her husband, her ADLs were mildly impaired.

#### 3.2.1. Neuropsychological Assessment

Based on her educational and vocational history, ML’s premorbid intellectual ability was estimated to be average. She scored 81/100 on the ACE-III, which was below established cut-off scores for normal performance (normal > 88; Table 1). Formal neuropsychological testing revealed a primary impairment in expressive language. Qualitatively, her speech was markedly dysfluent, and contained articulatory and occasional grammatical errors. Single-word repetition and verbal fluency were extremely impaired on testing. Her other language abilities (comprehension, semantic knowledge) and writing, however, remained preserved (Table 1, Figure 1). Indeed, she could provide a reliable history of her difficulties by writing her responses. Performance on verbal short-term and working memory measures were also reduced, but likely due to her dysfluent speech (Table 2, Figure 2). Visuospatial short-term and working memory, on the other hand, was sound (Figure 3). Encoding and retention of verbal and visual information was preserved. While basic visuo-perceptual abilities were intact, ML had subtle visuo-constructional difficulties evidenced by an imprecise and slightly disorganised copy of the Rey Complex Figure (Figure 4). Other aspects of executive functioning (rapid set-shifting, inhibitory control) as well as psychomotor speed were impaired.

#### 3.2.2. Clinical Opinion about the Patient’s Verbal and Visuospatial Short-Term and Working Memory Profile

ML’s profile is characteristic of nfv-PPA. Her verbal short-term and working memory span and sentence repetition were markedly impaired; however, frank motor speech deficits largely contributed to her impaired performance (Table 1 and Table 2). Notably, ML had greater difficulty with repeating sentences and multisyllabic words or phrases than (predominantly monosyllabic) digits. In contrast, her visuospatial short-term and working memory performance appeared relatively intact (Figure 3).

#### 3.2.3. Brain MRI and Clinical Diagnosis

The T1 coronal brain MR images revealed mild generalised cortical atrophy with particular involvement of the left peri-insular region anteriorly (Figure 5). Cerebral atrophy over the convexity was also present with widening of the interhemispheric fissure. The pattern of brain atrophy, clinical history, language, and neuropsychological profile were consistent with a diagnosis of nfv-PPA (Table 3b). Pathological confirmation was unavailable as ML remains alive.

### 3.3. sv-PPA Patient: JC

At presentation, JC was a 62-year-old, right-handed man (Table 1). He completed 16 years of education and worked as a principal of a primary school before retiring 2 years prior to the visit. His medical history was significant for a parathyroid cancer 13 years prior which was treated with a thyroidectomy and subsequently managed with levothyroxine, and ischaemic heart disease, with a myocardial infarction and 4-vessel coronary artery bypass graft surgery 4 years prior to the assessment. At the time of the assessment, JC was on antiplatelet and cholesterol-lowering medications. There was no report of symptoms of depression, anxiety, or stress on a self-report measure of recent mood and there were no significant periods of mood disorder noted. He demonstrated appropriate emotional reactivity during the assessment. There was no known family history of dementia.

JC was assessed following a 6-year history of speech and language difficulties with initial symptoms including forgetfulness and difficulties learning students’ names at work. He reported progressive difficulties recalling names of people and objects (i.e., plants, animals) in the past 3 years, as well as a decline in his language comprehension and semantic knowledge. He was reportedly an avid reader previously though this had declined due to difficulties understanding the meaning of words. No issues with reading the actual words or recognising letters were reported. According to JC’s wife, there was no change in behaviour or personality, eating habits, appetite, or weight. JC remained physically well and had no weakness or motor dysfunction. According to his wife, his ADLs were mildly impaired.

#### 3.3.1. Neuropsychological Assessment

Based on his education and vocational history, JC’s premorbid intellectual ability was estimated to lie within the average to high average range. He scored 67/100 on the ACE-III which was well below normal limits (normal > 88, Table 1). Qualitatively, his speech output was fluent with no phonological errors or substitutions, though occasional word-finding problems were noted, and he was slightly circumlocutory. Formal neuropsychological testing revealed intact visuospatial short-term and working memory (Table 1, Figure 3). Whilst his verbal working memory for numerical information and single words was intact, sentence repetition was compromised (Figure 2, Table 2). In terms of his language, JC demonstrated impaired confrontation naming, single-word comprehension and semantic knowledge; single-word repetition and verbal fluency, however, remained intact (Figure 1). Aside from suboptimal set-shifting, no significant executive functioning impairments were evident. JC’s visuo-constructional planning and organisation remained intact. No visual memory deficits were apparent (Figure 4); detailed assessment of JC’s verbal learning and memory, however, was not conducted on this occasion.

#### 3.3.2. Clinical Opinion about the Patient’s Verbal and Visuospatial Short-Term and Working Memory Profile

Overall, JC’s cognitive profile is consistent with the characteristic sv-PPA profile. Whilst his short-term and working memory for digits, words and visuospatial information remained relatively spared, his repetition of complex sentences was more problematic. Notably and consistent with his intact basic verbal short-term memory span, qualitative appraisal of his sentence repetition (Table 2) suggests that his poor performance results from inclusions of grammatically correct but superfluous words (see Discussion).

#### 3.3.3. Brain MRI and Clinical Diagnosis

T1 coronal brain MR images showed bilateral atrophy in the temporal lobes (left more markedly than right) particularly affecting the left hippocampal region and peri-insular region. Mild atrophy of the frontal lobes bilaterally was also evident (Figure 5). T2 weighted MRI images revealed evidence of scattered white matter hyperintensities in both hemispheres in keeping with small vessel ischaemic change. No established territorial infarcts were evident. The pattern of brain atrophy, clinical history, language, and neuropsychological profile were in keeping with a diagnosis of sv-PPA (Table 3c). JC underwent a Pittsburgh compound B (PiB) positron emission tomography scan (PiB-PET), which uses a radio-ligand of amyloid protein as a biomarker for AD [26]. The patient showed a low uptake of the PiB tracer, suggesting the absence of underlying Alzheimer pathology [26].

### 3.4. Typical AD Patient: TN

TN presented as a right-handed 67-year-old male. He completed nine years of formal education and worked in the government services for three years before working in hospitality and owning a business. At the time of assessment, he had been retired for 4 years. His medical history included ischaemic heart disease, with coronary artery bypass graft surgery 19 years prior to the assessment. TN also had a history of post-traumatic stress disorder arising from his previous employment, though this was well-managed at the time of assessment. He did not report significant symptoms of depression, anxiety, or stress on a self-report measure of recent mood. Regular medications included telmisartan for hypertension, antiplatelet medication, and selective serotonin reuptake inhibitors (SSRIs) for depressive symptoms. There was no known family history of neurodegenerative disease.

TN presented for assessment following a 7-year history of insidious memory decline, which had worsened considerably in the last 3 years. Both TN and his wife reported difficulties with his memory, specifically with names and topographical memory. Some organisational and planning difficulties were also noted which impacted his instrumental ADLs. TN’s wife had noticed mild apathy but there were otherwise no other personality or behavioural changes.

#### 3.4.1. Neuropsychological Assessment

Based on his education and vocational history, TN’s estimated premorbid intellectual ability fell in the average range. He scored 68/100 on the ACE-III, which was well below normal limits (normal > 88; Table 1). Consistent with his diagnosis, TN demonstrated prominent verbal and visual memory impairment on testing. He was unable to learn a word list over repeated trials, and his recall of a previously copied two-dimensional complex geometric figure after a 3-minute delay was extremely poor (Figure 4). Whilst verbal short-term and working memory was intact, visuospatial short-term memory was reduced, and his visuospatial working memory (i.e., Spatial Span Backward) was borderline-impaired (Table 1). Other executive functioning abilities were variable—he was extremely slow and made several errors on a set-shifting task. Basic visuo-perceptual skills and visuo-constructional abilities (e.g., drawing simple objects) were preserved, although some higher-level visuo-constructional difficulties were present (Figure 4). Psychomotor speed was intact. Finally, no overt expressive language issues were noted in conversation although confrontation naming and comprehension (i.e., word-picture matching) were below expectations on testing (Figure 1). Other aspects of language (repetition, verbal fluency, and higher-level semantic knowledge) were relatively intact.

#### 3.4.2. Clinical Opinion about the Patient’s Verbal and Visuospatial Short-Term and Working Memory Profile

TN’s verbal and visuospatial span profile was consistent with typical AD (Figure 3). As expected from a typical AD diagnosis, TN’s visuospatial working memory ability (as demonstrated on Spatial Span Backward) was poor, whilst basic verbal and visuospatial short-term memory span remained relatively preserved.

#### 3.4.3. Brain MRI and Clinical Diagnosis

T1 coronal brain MR images revealed atrophy of the medial temporal region bilaterally including the hippocampus, as well as diffuse frontal and parietal atrophy (Figure 5). Ventricular enlargement was also evident. T2 weighted MRI images revealed scattered white matter hyperintensities in both hemispheres in keeping with small vessel ischaemic change. No established territorial infarcts were evident.

Two years after his assessment, TN underwent in vivo amyloid-PET imaging, which showed uptake of the amyloid ligand above the cut-off for an amyloid based pathology, indicating the presence of underlying Alzheimer disease [26]. The clinical history, language, neuropsychological profile, and confirmation of underlying Alzheimer pathology were consistent with a clinical diagnosis of typical AD [27].

#### 3.4.4. Summary of the Short-Term and Working Memory Profiles across Patients and Relative to a Matched Control Group

**Figure 6:**
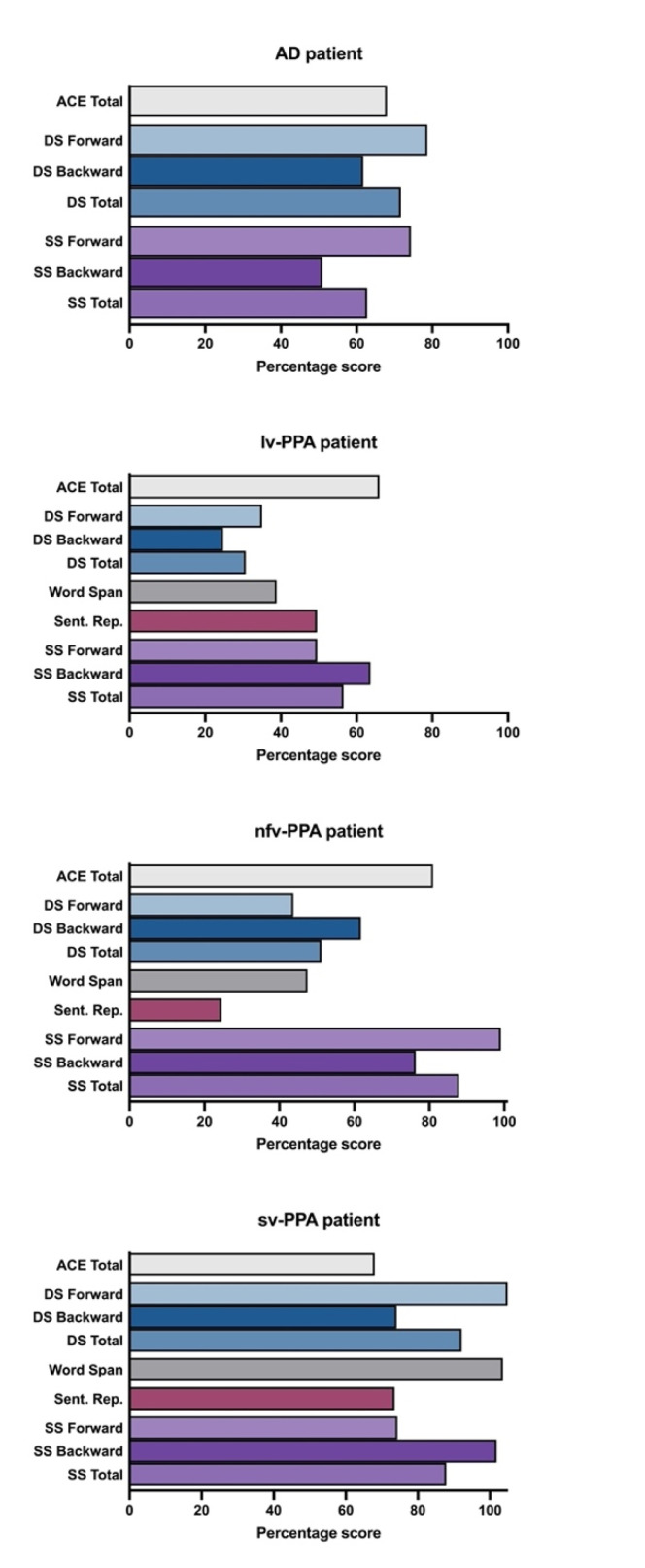
Short-term and working memory performances represented as percentage scores of normal performance (i.e., the patient’s score divided by a control mean, times by 100). The ACE total bar charts are the patient’s raw score on this measure. Control means taken from Foxe, Irish [4]. Notes, ACE: Addenbrooke’s Cognitive Examination-III; DS: Digit Span; Sent. Rep: Sentence Repetition; SS: Spatial Span.

These short-term and working memory profiles were established based on standardised norms from various population groups and sizes: WAIS-III and WMS-III Digit and Spatial Span [17,22], and Word Span and Sentence Repetition norms [4]. To ensure that our findings were not due to differences across normative populations, performance profiles of the case vignettes were compared to one sample of Australian matched controls [4] and are displayed as percentage scores (Figure 6).

Inspection of these scores confirm that the lv-PPA and nfv-PPA patients were disproportionately impaired on Digit and Word Span relative to Spatial Span and overall cognitive ability (i.e., ACE total). By contrast, performance differences across verbal and visuospatial modalities were less evident for the sv-PPA and AD patients. Sentence Repetition performance across patients and within individual performance profiles was variable and uninterpretable across patients based on raw scores alone.

Regarding the specific short-term and working memory profiles, the lv-PPA, sv-PPA and AD patients demonstrated greater impairment on the Digit Span Backward than Forward tasks compared to controls. The reverse pattern was found for the nfv-PPA patient. These findings suggest that, over and above the inherent general difficulty associated with verbal working memory, the lv-PPA, sv-PPA and AD patients displayed more difficulty on this task relative to their verbal short-term memory capacity. As previously discussed, the nfv-PPA patient’s impoverished speech is likely to have contributed to their performance on this task.

Overall Spatial Span profiles were distinct across patients. Relative to overall cognitive ability (i.e., ACE Total), the sv-PPA and nfv-PPA patients’ Spatial Span performance was relatively spared. By contrast, the lv-PPA and AD patient’s Spatial Span performance was reduced somewhat. These findings were in keeping with other measures of visuospatial episodic memory (i.e., RCFT 3-minute recall) and visuo-construction (RCFT Copy).

## 4. Discussion

While significant advancements have been made in the classification of PPA and its variants, challenges remain in clinical practice in differentiating PPA profiles in individual patients. Using case vignettes, we demonstrate that the canonical phonological disturbance displayed in lv-PPA is distinguishable from other PPA language profiles when using a selection of basic language and short-term memory measures. Importantly, we highlight how speech output and language deficits differentially interfere with verbal memory performance across the PPA variants, and how these differences provide insights into the underlying cognitive processes affected in these syndromes. Further, we demonstrate that visuospatial span tasks are essential for the assessment of PPA as they measure memory capacity without language contamination.

The lv-PPA patient demonstrates the typical verbal short-term memory deficit observed in this syndrome: very impaired digit and word span, and markedly reduced sentence repetition, in the context of relatively spared repetition of single two- and three-syllabic words [1,2]. Importantly, this occurred in the absence of frank motor speech deficits. Phonological paraphasias were, however, present. In contrast the nfv-PPA patient also performed poorly on verbal repetition tasks, but this performance was contaminated by frank motor speech deficits. Notably, and consistent with previous studies, we found that the nfv-PPA patient’s verbal short-term memory performance declined as the motor speech sequencing requirements increased (i.e., repeating monosyllabic digits compared to a span of multisyllabic words and/or phrases [3]). Considering the sv-PPA patient’s profile, we found digit and word span were spared whereas sentence repetition was compromised, likely to be due to his degraded semantic store [3]. Specifically, it is thought that the dissolving semantic knowledge and ability to form conceptual representations in sv-PPA may impact on the capacity to ‘chunk’ verbal material into meaningful components—a skill necessary for holding larger quantities of verbal information [3,11,28]. While not systematically verified in the current study, the sv-PPA patient’s occasional circumlocutory responses and/or word inclusions on the Sentence Repetition task would support this interpretation.

Taken together, we propose that, when assessing for lv-PPA, Digit Span Forward and Word Span tasks are more robust measures of verbal short-term memory than Sentence Repetition or Digit Span Backward tasks, as the former tasks are less susceptible to language and/or dysexecutive contamination [4,11,12]. This observation notwithstanding, important qualitative information can be gained from Sentence Repetition that will help with the distinction between the two non-fluent PPA syndromes; specifically, its ability to elicit phonological paraphasias (in lv-PPA) or motor speech deficits (in nfv-PPA) [11,12]. For the assessment of sv-PPA, we caution that some patients may perform poorer on Sentence Repetition than other span tests as their degraded semantic knowledge may preclude their ability to ‘chunk’ and/or form meaningful representations in mind [3,11,28].

In contrast to their similar verbal span and sentence repetition performance, the lv-PPA and nfv-PPA patients showed distinct visuospatial span profiles. The disproportionately compromised visuospatial short-term and working memory in lv-PPA relative to the other PPA variants is consistent with a growing body of research and alludes to their distinct neuroanatomical profile [4,29,30,31,32]. Briefly, brain regions involved in visuospatial short-term and working memory, including bilateral posterior temporal and parietal brain structures, are more compromised in lv-PPA and AD than in nfv-PPA and sv-PPA [4,33,34,35]. Undoubtedly, and unlike in nfv-PPA where the deficit remains primarily verbal, the impaired short-term memory of lv-PPA extends to the non-verbal domain, even at low levels of difficulty [30,31,36,37]. Based on these findings, we propose that when assessing PPA patients without motor features, difficulty on basic/lower-load visuospatial short-term memory tests strongly indicates lv-PPA [31,38,39]. This distinction will assist the diagnostic process, particularly in the presence of a mixed language profile.

It is notable that short-term and working memory deficits are not limited to PPA. Indeed, we found that the AD patient showed impairments across these domains. Unlike the PPA patients, however, the AD patient’s overall visuospatial span was significantly worse than their overall verbal span profile. These findings are consistent with the commonly held view that multiple components of visuospatial memory, including processing, integration, storing, and retaining visual material, break down in the earlier stages of typical AD [4,31,33,40]. Consistent with this view, the visuospatial difficulties of the AD patient extended beyond Spatial Span—with deficits also noted on complex visuo-constructional, visuospatial episodic memory, and attentional tasks (i.e., Rey Complex Figure, Trails A and B). Importantly, the widespread memory and visuospatial deficits of the AD patient supports the opinion that AD is distinct from lv-PPA [31,41]. That is, lv-PPA is not simply typical AD with additional language deficits [42]. While this nuanced distinction may seem unnecessary, it has clinical implications when addressing the care needs, treatment options, and estimated survival of either AD or lv-PPA [43]. To that end, we propose requisite assessment of both verbal and visuospatial cognitive domains for the differential diagnosis of AD and lv-PPA.

In clinical practice, awareness that the cognitive profiles of PPA and AD vary across individuals is vital. Most studies compare matched PPA subgroups at a single point in time (typically at the mild to moderate disease severity stage) and provide findings which typically overemphasise the differences across variants but underemphasise the differences within each syndrome. Studies that have investigated within syndromes, however, suggest that the language and cognitive profile of lv-PPA varies considerably [6,7,8,9,10,44]. For example, it is reported that while most lv-PPA patients present with multi-domain cognitive impairment at baseline assessment, a subset of lv-PPA patients present with relatively circumscribed language deficits with relatively mild cognitive deficits in other domains [6]. Important to this topic is the awareness that, with disease progression, language and cognitive abilities of PPA and AD inevitably decline, eventuating in a manifold dissolution of functional abilities [45,46,47]. As such, the distinct cognitive profiles observed in the earlier stages of these diseases may become less apparent in later disease stages. To illustrate this point, Table 4 demonstrates the decline of Spatial and Digit Span performance across PPA and AD patients, stratified by overall cognitive ability. Put together, clinicians should take a gestalt approach to assessing PPA and AD in clinical practice and consider the ‘moving parts’ of language and cognitive deficits, as well as overall cognitive ability, before forming a formal clinical diagnosis.

Several caveats warrant attention. Pathological confirmation was not available in any of the PPA cases. Nonetheless, the clinical, cognitive, and imaging information provided in the current study is typical of what is commonly available at baseline assessment in routine clinical practice (i.e., non-tertiary/specialised centres) and was sufficient to establish a clinical diagnosis of each PPA variant [1]. It is reported that ~70% of lv-PPA patients have underlying Alzheimer pathology, ~70% of nfv-PPA have tauopathy, and ~85% of sv-PPA have TDP−43, and that detailed and careful clinical, cognitive, and imaging examination improves this pathological correspondence [48,49,50]. More research, however, is warranted to determine if specific cognitive profiles within PPA syndromes can further discern the pathological course.

The nfv-PPA patient in this study demonstrated a severe motor speech disorder with severe articulatory and prosodic impairment. We are aware, however, that other nfv-PPA language profiles exist (e.g., agrammatism and articulatory impairment without motor speech problems). Of particular interest to our team is the extent to which nfv-PPA language profiles without motor speech problems interfere with verbal short-term memory measures. Future research is needed to determine the extent to which verbal short-term memory performance is compromised across the distinct nfv-PPA language profiles.

Lastly, a proportion of nfv-PPA patients will develop Parkinsonian features (i.e., limb apraxia, akinesia/bradykinesia, motor rigidity) as the disease progresses (typically in the moderate to severe disease stages) [51]. In the current study, we do not refer to these patients as we assumed that they are more easily distinguishable from lv-PPA based on their clinical profiles alone (as Parkinsonian features are not common in lv-PPA) [51]. We acknowledge, however, that nfv-PPA patients with Parkinsonian features are likely to perform poorly on visuospatial related tasks due to their inherent motor dysfunction [38,39]. We therefore advise that our findings are only applicable to nfv-PPA without Parkinsonian features. Future research is warranted to delineate the nfv-PPA visuospatial short-term and working memory profiles with or without concomitant Parkinsonian features.

## 5. Conclusions

In summary, using case vignettes, we demonstrate the canonical verbal short-term memory profile of lv-PPA and how it differs from the other PPA variants as well as typical AD. Importantly, we demonstrate that a combination of verbal short-term and working memory measures commonly used in clinical settings can provide crucial information regarding the cognitive mechanisms underlying language disturbances across PPA variants. Further, we demonstrate that visuospatial span tasks are essential for the assessment of PPA as they measure memory capacity without contamination of language ability.

## Figures and Tables

**Table 1:** Demographics and neuropsychological test scores.

Domain	Cognitive Test	Subtest (Max Score)	TN: AD Patient	NS: lv-PPA Patient	ML: nfv-PPA Patient	JC: sv-PPA Patient
**Demographics**						
Sex (m:f)			Male	Male	Female	Male
Age (y)			67	66	64	62
Handedness			Right	Right	Right	Right
Education (y)			9	12.25	12	16
Disease duration (y)			7.4	3.5	6.6	5.4
**General cognition** **Attention and executive functioning**	ACE-III	Total (100)	68 **	66 **	81 *	67 **
	Trails	A time (errors)	48 (0)	56 (0) *	65 (0) **	43 (0)
		B time (errors)	365 (3) **	460 (2) **	344 (0) **	99 (0)
		B-A time difference	317 **	404 **	279 **	56
	Letter fluency	F, A, S	36	14 **	12 **	36
**Short-term and working memory**	Digit Span	Raw Forward (longest)	9 (6)	4 (3) **	5 (4) *	12 (8)
		Raw Backward (longest)	5 (4)	2 (3) **	5 (4) *	6 (4)
		Raw Total (SS)	14 (9)	6 (4) **	10 (6) *	18 (11)
	Spatial Span	Raw Forward (longest)	6 (4)	4 (3) *	8 (6)	6 (6)
		Raw Backward (longest)	4 (4) *	5 (4)	6 (4)	8 (6)
		Raw Total (SS)	10 (6) *	9 (5) *	14 (10)	14 (10)
	Sentence Rep	Raw Total (14)	Nil	6 **	3 **	9 **
	Word Span	Raw Total (30)	Nil	9 **	11 **	24
**Memory**	RCFT	Copy (36)	12.5 **	29 *	30 *	36
		3-min recall (36)	1 **	7.5 *	18.5	22.5
**Language**	SYDBAT	Naming (30)	23 *	20 **	30	10 **
		Repetition (30)	30	24**	2 **	30
		Comprehension (30)	25 *	28	30	20 **
		Semantic Assoc. (30)	27	29	29	19 **
**Visuospatial**	Clock drawing	(5)	5	5	5	5
	ACE Visuospatial	(16)	15	15	14	16
	RCFT	Copy time (secs)	594 **	513 **	229	290
**Mood**	DASS-21	Depression	4 (Normal)	0 (Normal)	3 (Normal)	0 (Normal)
		Anxiety	2 (Normal)	1 (Normal)	3 (Normal)	0 (Normal)
		Stress	4 (Normal)	4 (Normal)	4 (Normal)	3 (Normal)
**Functional capacity**	FRS	Total Rasch	0.16 (Mod.)	2.86 (Mild)	5.39 (V. Mild)	2.19 (Mild)
	CDR-FTLD	Sums of boxes (SoB)	5 (Mild)	1.5 (Quest.)	4 (V. Mild)	2 (Quest.)

Notes: ACE-III: Addenbrooke’s Cognitive Examination-Third Edition [13]; ACE Visuospatial: Visuospatial sub-score of the Addenbrooke’s Cognitive Examination-III; CDR-FTLD: Frontotemporal Lobar Degeneration-Modified Clinical Dementia Rating Scale [14]; Clock drawing: Clock drawing subtest of the Addenbrooke’s Cognitive Examination-III; DASS–21: Depression Anxiety Stress Scale–21 items [15,16]; Digit Span: Digit Span subtest of the Wechsler Adult Intelligence Scale–III WAIS-III; [17]; FRS: Frontotemporal Dementia Rating Scale [18]; Letter Fluency: Letters F, A and S [19]; Mod.: Moderate; Quest.: Questionable; RCFT: Rey Complex Figure Test [20]; Secs: seconds; Sentence Repetition: Sentence Repetition from the Multilingual Aphasia Examination [21]; Spatial Span: Spatial Span subtest of the Wechsler Memory Scale–III (WMS–III) [22]; SS: scaled score; SYDBAT: The Sydney Language Battery [23]; Trails: Trail Making Test [24]; V. Mild: Very Mild; Word Span: word span test from Leyton, Savage [3]. * indicates borderline performance: 1.3 < z-score < 2.0; 3 < percentile < 9; ** indicates extremely low performance: z-score < −2.0; percentile < 2.

**Table 2:** PPA patients’ responses on the Multilingual Aphasia Examination Sentence Repetition test.

		Analysis on Sentence-Level						Analysis on Word-Level							
		***0 = No; 1 = Yes***						***Frequency***							
**Item**	**Produced Sentence**	**Correct 0/1**	**Erroneous Correct 0/1**	**Uncertain/Repaired Correct 0/1**	**Required Stimulus Repetition Correct 0/1**	**Misordered Words Correct 0/1**	**Violation of Syntactic Rules Correct 0/1**	**Omission**	**Semantic Substitution**	**Formal Errors**	**Phonological Errors**	**Phonological Errors Affecting Morphemes**	**Grammatical Errors**	**Sound Deviations**	**Not Classified Errors**
**NS: lv-PPA patient**															
1	Take this home	1	0	0	0	0	0								
2	Where is the child?	1	0	0	0	0	0								
3	The car will not run	1	0	0	0	0	0								
4	Why are they not living here?	1	0	0	0	0	0								
5	The band (.) played and the /kraʊnd/ (5) cheered	0	1	0	0	0	0				1				
6	Where are you going to work next summer?	1	0	0	0	0	0								
7	He sold his house /ən/ moved to the farm	0	1	0	0	0	0	1							
8	Work in the garden until you’ve picked all the beans	1	0	0	0	0	0								
9	The artist /peɪtəd / painted (3) many pictures of the /fɑ: / no sorry	0	1	0	0	0	0	4	1		2				
10	This doctor doesn’t go to all of the towns	0	1	0	0	0	0	3	1 *						
11	She should be able to tell us when /ʃɜ: / (2) when she (.) is (.) performing	0	1	0	0	0	0	4		1					
12	Why /dɒn / (.) that group (1) why doesn’t that group apply (.) for (.) money	0	1	0	0	0	0	8	1 *		1				
13	Many /pi:pəl / (5) they were not able to get work because of the (.) weather	0	1	0	0	0	0	6	2 *				1		
14	*Did not attempt*														
	Total	6	7	0	0	0	0	26	5	1	4	0	1	0	0
**ML: nfv-PPA patient**															
1	/teɪki ðə/	0	1	0	0	0	0	1							
2	/wɜ:ʳɪz ðə/ child	1	0	0	0	0	0								
3	/kɑ (2) ðə kɑ wɪl wuz nɒʔ rʌn/	0	1	0	0	0	0		2 **						
4	why /ɑ neɪ nɒʔ/ living here	1	0	0	0	0	0								
5	/ðɜ:ʳ beɪn peɪʔ ə ən tʃi (4) tʃeəʳz/	0	1	0	0	0	0	1							
6	/wɜ ə ju: goʊɪŋ tu: wɜ:ʳʔ nəʔ zə nə nətʃə (3) zʌmə/	1	0	0	0	0	0								
7	/ i: (.) sʊld ɪz haʊs əndə ðə wi: (4) fɑm/	0	1	0	0	0	0	3							
8	/wɜ:ʳk ən ðə gɑ:dən æn (4) bɪk tʃɒ gɒ ðə bi:nz/	0	1	0	0	0	0	4							
9	/ðɜ:ʳ ɑ:dɜ:ʳzd peɪʔ (3) / um / peɪndəd/… no	0	1	0	0	0	0	8							
10	/ ɪz (3) ɒl ðɜ:ʳ kʊntri: (1) ɒl ðɜ:ʳ (1) dɒz ɪn ðə kʊntri:/	0	1	0	0	0	0	7							
11	/i: wɒz/ no	0	1	0	0	0	0	13							
12	/waɪz du ðə gruf (3) waɪʔs (4) wəz/	0	1	0	0	0	0	10							
13	*Did not attempt*														
14	*Did not attempt*														
	Total	3	9	0	0	0	0	47	2	0	0	0	0	0	0
**JC: sv-PPA patient**															
1	Take this home	1	0	0	0	0	0								
2	Where is the child?	1	0	0	0	0	0								
3	The car will not run	1	0	0	0	0	0								
4	Why are they not living here?	1	0	0	0	0	0								
5	The band played and the crowd cheered	1	0	0	0	0	0								
6	Where are you going to work next summer?	1	0	0	0	0	0								
7	He sold his house and they moved to the farm.	1	0	0	0	0	0								
8	Work in the garden until you have picked all the beans	1	0	0	0	0	0								
9	The artist painted many of the beautiful scenes in this valley	1	0	0	0	0	0								
10	This doctor does not travel to all the towns in this country	0	1	0	0	0	0		1						
11	He should actually be able to tell us when she will actually be performing here	0	1	0	0	0	0		2 **						
12	Why do members of that group never write to their representatives of their group for aid?	0	1	0	0	0	0		3 **						
13	Many men and women were not able to get to their work because of the severe snowstorm	0	1	0	0	0	0		1 **						
14	The members of the committee have agreed to hold their meeting on the first Tuesday of every month	0	1	0	0	0	0		1						
	Total	9	5	0	0	0	0	0	8	0	0	0	0	0	0

Notes, Sentences were transcribed using the international phonetic transcription (IPA). Sentences were scored using the Hohlbaum, Dressel [12] scoring criteria. Notes, ʔ: glottal stop; (2) represents pause in seconds; (.) represents pause of <1 s; *: These errors are coded as semantic substitutions but in fact they do not appear to arise as a result of impaired lexical retrieval. Rather the lv-PPA patient decodes the meaning but cannot repeat the content word by word. So, he paraphrases, e.g., people for men and women; **: These errors are additions. The Hohlbaum system codes them as semantic substitutions, although there is no specific code for such errors.

**Table 3:** (**a**–**c**) PPA patients according to the Gorno-Tempini, Hillis [1] international consensus criteria of primary progressive aphasia.

(a)	Diagnostic Criteria for Logopenic Variant of PPA	Patient NS	(b)	Diagnostic Criteria for Non-Fluent Variant of PPA	Patient ML	(c)	Diagnostic Criteria for Semantic Variant of PPA	Patient JC
	**I. Clinical diagnosis of logopenic variant PPA**			**I. Clinical diagnosis of non-fluent/agrammatic variant PPA**			**I. Clinical diagnosis of semantic variant PPA**	
	**Both of the following core features must be present:**			**At least one of the following core features must be present:**			**Both of the following core features must be present:**	
	1. Impaired single-word retrieval in spontaneous speech and naming	*✓*		1. Agrammatism in language production	*✓*		1. Impaired confrontation naming	*✓*
	2. Impaired repetition of sentences and phrases	*✓*		2. Effortful, halting speech with inconsistent speech sound errors and distortions (apraxia of speech)	*✓*		2. Impaired single-word comprehension	*✓*
	**At least 3 of the following other features must be present**:			**At least 2 of 3 of the following other features must be present**:			**At least 3 of the following other features must be present**:	
	1. Speech (phonologic) errors in spontaneous speech and naming	*✓*		1. Impaired comprehension of syntactically complex sentences	Unknown		1. Impaired object knowledge, particularly for low-frequency or low-familiarity items	*✓*
	2. Spared single-word comprehension and object knowledge	*✓*		2. Spared single-word comprehension	*✓*		2. Surface dyslexia or dysgraphia	*✓*
	3. Spared motor speech	*✓*		3. Spared object knowledge	*✓*		3. Spared repetition	*✓*
	4. Absence of frank agrammatism	*✓*		**II. Imaging-supported non-fluent/agrammatic variant diagnosis**			4. Spared speech production (grammar and motor speech)	*✓*
	**II. Imaging-supported logopenic variant diagnosis**			**Both of the following criteria must be present**:			**II. Imaging-supported semantic variant diagnosis**	
	**Both of the following criteria must be present**:			1. Clinical diagnosis of non-fluent/agrammatic variant PPA	*✓*		**Both of the following criteria must be present:**	
	1. Clinical diagnosis of logopenic variant PPA	*✓*		2. Imaging must show one or more of the following results:			1. Clinical diagnosis of semantic variant PPA	*✓*
	2. Imaging must show at least one of the following results:			a. Predominant left posterior fronto-insular atrophy on MRI or	*✓*		2. Imaging must show one or more of the following results:	
	a. Predominant left posterior perisylvian or parietal atrophy on MRI	*✓*		b. Predominant left posterior fronto-insular hypoperfusion or hypometabolism on SPECT or PET	Not available		a. Predominant anterior temporal lobe atrophy	*✓*
	b. Predominant left posterior perisylvian or parietal hypoperfusion or hypometabolism on SPECT or PET	Not available		**III. Non-fluent/agrammatic variant PPA with definite pathology**			b. Predominant anterior temporal hypoperfusion or hypometabolism on SPECT or PET	Not available
	**III. Logopenic variant PPA with definite pathology**			**Clinical diagnosis (criterion 1 below) and either criterion 2 or 3 must be present:**			**III. Semantic variant PPA with definite pathology**	
	**Clinical diagnosis (criterion 1 below) and either criterion 2 or 3 must be present**:			1. Clinical diagnosis of non-fluent/agrammatic variant PPA	*✓*		**Clinical diagnosis (criterion 1 below) and either criterion 2 or 3 must be present:**	
	1. Clinical diagnosis of logopenic variant PPA	*✓*		2. Histopathologic evidence of a specific neurodegenerative pathology (e.g., FTLD-tau, FTLDTDP, AD, other)	Not available		1. Clinical diagnosis of semantic variant PPA	*✓*
	2. Histopathologic evidence of a specific neurodegenerative pathology (e.g. AD, FTLD-tau, FTLD-TDP, other)	Not available		3. Presence of a known pathogenic mutation	Not available		2. Histopathologic evidence of a specific neurodegenerative pathology (e.g., FTLD-tau, FTLDTDP, AD, other)	Not available
	3. Presence of a known pathogenic mutation	Not available					3. Presence of a known pathogenic mutation	Not available

Notes, Abbreviations: AD = Alzheimer’s disease; FTLD = frontotemporal lobar degeneration; PPA = primary progressive aphasia; TDP = TAR DNA-binding protein.

**Table 4:** Means of Spatial and Digit Span performances stratified by ACE-III Total performance. Sample PPA population was taken from Foxe, Irish [4].

ACE-III Total Score			<40	40–49	50–59	60–69	70–79	80–89	90–100
AD	Spatial Span	Forward	3.2	3.6	4.5	4.9	6.8	5.1	-
		Backward	1.0	2.2	3.7	3.2	5.5	4.8	-
	Digit Span	Forward	4.2	6.5	8.7	8.1	8.4	8.7	-
		Backward	1.6	2.9	3.7	3.7	5.1	5.3	-
lv-PPA	Spatial Span	Forward	4.8	4.3	4.0	5.6	6.8	8.0	9.0
		Backward	3.0	4.5	2.7	5.4	6.4	7.7	8.0
	Digit Span	Forward	4.0	5.0	5.7	5.9	8.4	9.3	9.0
		Backward	2.3	2.0	2.3	4.0	4.6	6.3	8.0
nfv-PPA	Spatial Span	Forward	5.0	-	5.0	6.0	8.5	7.6	7.0
		Backward	4.0	-	2.5	3.5	6.0	6.7	8.0
	Digit Span	Forward	-	-	5.0	6.0	6.3	7.5	10.0
		Backward	2.0	-	2.0	3.0	4.2	4.8	5.7
sv-PPA	Spatial Span	Forward	6.8	7.3	8.5	8.1	8.4	9.4	8.0
		Backward	6.5	4.7	7.8	8.1	8.8	9.2	9.0
	Digit Span	Forward	8.5	11.0	9.0	9.7	12.2	12.6	8.0
		Backward	5.8	5.7	5.3	5.8	8.0	8.2	8.0

Note, this sample population did not include nfv-PPA patients that scored between 40–49 or AD patients that scored between 90–100 on the ACE-III.

## Data Availability

The datasets generated during and/or analysed during the current study are available from the corresponding author on reasonable request. No part of the study procedures or analyses were preregistered prior to the research being undertaken. The Addenbrooke’s Cognitive Examination-Third edition (ACE-III) and Sydney Language Battery (SYDBAT) are freely available at https://frontierftd.org (accessed on 11 August 2021). Legal copyright restrictions prevent public archiving of the other neuropsychological tests used in this research. These materials can be obtained from the copyright holders in the cited references.

## Appendix A

Administration and scoring guidelines of the SYDBAT can be found at https://frontierftd.org (accessed on 11 August 2021). For the administration of the Naming subtest, the examiner is required to display the item/picture and ask the participant “What is this called?”. The participant is encouraged to give their response in a timely manner but is not penalised for a delayed response. In accordance with the SYDBAT administration guidelines, the examiner is required to prompt the patient in these circumstances: (i) if the participant provides an abbreviated version of the word (e.g., “bike”, “PC”), the examiner should say “What is the full name?”; (ii) if the participant provides a response that is vague or describes the item (e.g., “oh that’s a type of food”), the examiner should say “Can you give me the exact name?”; (iii) if the participant uses an alternative or colloquial version (e.g., “wireless” rather than “radio” for Item 8), the examiner should say “Do you know another word for that?” or “What is the formal name for that?”; (iv) if the participant grossly misperceives an item, the examiner should clarify it. For example, for Item 25 (Tiara), some participants do not recognise that it sits on top of someone’s head. The examiner should clarify and say, “This is on someone’s head”. For another example, for item 28 (balaclava), if the participant responds with “robber”, the examiner should say “But what is the name of the thing he is wearing?”. No other prompts are permitted for the SYDBAT Naming subtest. In this case study, the sv-PPA patient received two (iv) prompts, and the lv-PPA and nfv-PPA patient received one (i) prompt. It is strongly encouraged that participants provide a spoken/verbal response for the Naming subtest. Given the extent of the nfv-PPA patient’s speech output problems, however, it was decided that it was more clinically useful to test her ability to freely-recall the names of items without spoken language (i.e., in written form). To remain consistent with the scoring guidelines for spoken responses, the nfv-PPA patient was penalised for spelling errors. Written responses were also permitted for the ACE-III Orientation, Attention, Memory, and Naming items. For all other items requiring speech (i.e., ACE-III: Verbal Fluency, Repetition, Reading; Digit Span; Letter Fluency; Sentence Repetition; SYDBAT Repetition etc.), she was required to provide spoken/verbal responses.
